# Main Ingredient of Yinhua Pinggan Granules Combined with Meropenem Alleviated Lung Injury Induced by Multidrug-Resistant *Klebsiella pneumoniae* via Inhibiting NF-κB Pathway and NLRP3 Inflammasome Activation

**DOI:** 10.4014/jmb.2412.12014

**Published:** 2025-05-15

**Authors:** Shengyao Zhang, Haofang Wan, Xiaodan Guan, Daojun Yu, Jiehong Yang, Haitong Wan

**Affiliations:** 1 Biosafety Laboratory of Integrated Traditional Chinese and Western Medicine, School of Basic Medical Sciences, Zhejiang Chinese Medical University, Hangzhou 310053, P. R. China; 2 College of Traditional Chinese Medicine, Zhejiang Chinese Medical University, Hangzhou 310053, P. R. China; 3 Fuyang Research Institute of Zhejiang Chinese Medical University, Hangzhou 311400, P.R. China; 4 Hangzhou First People’s Hospital, Hangzhou 310003, P.R. China; 5 Academy of Chinese Medical Sciences, Henan University of Chinese Medicine, Zhengzhou, 450046, Henan Province, P. R. China

**Keywords:** Multidrug-resistant *Klebsiella pneumoniae*, Yinhua Pinggan granules, combination therapy, tmexCD1-toprJ, reactive oxygen species, NF-κB, NLRP3

## Abstract

In combating the global epidemic of multidrug-resistant *Klebsiella pneumoniae* (MDR-KP), combination therapy with the active ingredient of meropenem (MER) is gaining attention as a new therapeutic approach. In this study, the effect of OAY (orthogonal combination drug of active ingredients in YHPG) in combination with MER on MDR-KP was assessed using the microdilution technique. Additionally, the antimicrobial effect of OAY in combination with MER on MDR-KP was analyzed by reactive oxygen species (ROS), alkaline phosphatase (AKP), and RT-qPCR techniques. Furthermore, the expression levels of critical targets within the NF-κB/NLRP3 pathway were assessed via HE staining and western blot in an MDR-KP-infected mice model. Our results confirmed that the OAY-MER combinations inhibited MDR-KP biofilm formation. In the meantime, the compromise of membrane integrity led to the generation of ROS, which subsequently resulted in a decrease in the activity of intracellular enzymes, specifically AKP. We also found that the combination of OAY-MER reversed tmexCD1-toprJ-mediated MER resistance in MDR-KP. Finally, by a mouse model of MDR-KP infection, the data demonstrated that OAY and YHPG ameliorated lung injury and bacterial infections in the lungs, and significantly reduced NF-κB P-p65, NLRP3, and C-GSDMD protein expression in mouse lung tissues. The findings suggest that the combination of OAY with meropenem may have great potential for clinical application and could provide a theoretical basis for its use in treating MDR-KP infections.

## Introduction

*Klebsiella pneumoniae* (KP) possesses an extensive accessory genome that contributes to its ability to cause various infections [1]. Due to its extended antibiotic resistance and global spread via mobile genetic elements, multidrug-resistant *Klebsiella pneumoniae* (MDR-KP) has become an imminent threat in the clinical setting, leading to higher mortality [2, 3]. Meropenem (MER), a beta-lactam antibiotic, is used to treat a variety of infections, including sepsis and meningitis [4, 5]. Moreover, clinical treatment has become increasingly difficult for drug-resistant and hypervirulent strains of bacteria, hence the urgent need to find new strategies for MDR-KP.

Numerous signaling pathways contribute to the inflammatory response, with NF-κB recognized as an important component [6]. Activated NF-κB plays a significant role in modulating cell proliferation and apoptosis, as well as in the synthesis of inflammatory cytokines [6-8]. In addition, research has indicated the activated NF-κB may be an upstream regulator in inflammasome [6]. Inflammasomes are crucial components in the innate immune system, with NLRP3 inflammasome (which is comprised of NLRP3, ASC, and caspase-1), being the most extensively studied [9]. GSDMD undergoes cleavage by inflammatory caspases and demonstrates pore-forming capabilities that facilitate the process of pyroptosis [10, 11]. Another study showed that dynasore greatly inhibited the NF-κB signaling pathway, activated the NLRP3 inflammasome, leading to mitigated LPS-induced acute lung injury in a mice model [12]. Therefore, the NF-κB/NLRP3 pathway probably represents a novel therapeutic target for bacterial infections and lung injury.

The mechanisms of bacterial resistance to MER agents include drug efflux and the loss of porins, which are significant factors contributing to this resistance [13, 14]. TmexCD1-*toprJ1* was recently reported to confer clinical strains significant resistance and weaken the effectiveness of tigecycline (TIG) [15, 16]. The tmexCD-toprJ gene cluster underwent horizontal gene transfer between bacteria via plasmids, a mechanism that enabled the rapid spread of drug-resistant genes in bacterial populations [17]. However, it is not known whether the tmexCD1-*toprJ1* efflux pump mediates the efflux action of clinically important pathogens on MER. In addition, research had shown the generation of reactive oxygen species (ROS) contributed to the bacteriostatic effects of antibiotics [18]. An overabundance of ROS can induce lipid peroxidation, which diminishes the fluidity of the bacterial membrane and alters its properties. This alteration subsequently compromises the integrity of membrane proteins, ultimately resulting in bacterial cell death [19, 20]. Furthermore, research indicates that nearly all strains of KP are capable of biofilm formation, particularly those exhibiting elevated resistance rates [21-23]. Biofilm formation, one of the most important virulence features of KP, activates virulence factors and resists phagocytosis, thus facilitating the onset and development of bacterial infectivity and resistance [23, 24]. Moreover, biofilm formation significantly enhances the expression of efflux pumps compared to planktonic bacteria, resulting in heightened bacterial resistance to antibiotics [25-27].

Antimicrobial drug development has always been a challenge, as evidenced by the limited number of new antibiotics that have been successfully introduced into clinical practice [28, 29]. Consequently, the introduction of viable methods to expand treatment options is urgently required. Repurposing approved drugs as antibiotic adjuvants could well improve the effects of currently available antibiotics. Yinhua Pinggan granules (YHPG) are a traditional Chinese medicine that contains ingredients such as honeysuckle, tiger balm, *Pueraria lobata* (kudzu), and bitter amygdalin (Amy). We found that YHPG and its active ingredients, which include chlorogenic acid (CA), puerarin (Pue), Amy and polydatin (PD), restored the inhibitory activity against the MDR bacteria by facilitating the antibiotic's intra-bacterial accumulation [30-34]. In this research, the four main components were selected for orthogonal experiments, and we referred to our previous studies for the maximum harmless concentrations to the cells [31, 33, 35].

Consequently, we aimed in this study to find a promising therapeutic strategy for MDR-KP, and to elucidate MER in combination with the active ingredients of OAY/YHPG, as well as the mechanism by which the OAY/YHPG-MER combination acted against MDR-KP.

## Materials and Methods

### Reagents

CA, Pue, Amy, and PD were bought from the Chengdu Alfa Biotechnology (China). Crystal violet staining solutions were purchased from Beyotime (China). Alkaline phosphatase (AKP) kits were obtained from Beyotime. The 2X Universal SYBR Green Fast qPCR Mix was purchased from ABclonal (China), and ROS kits (Green Fluorescent) were bought from Biobying (China).

### Bacterial Strains and Culture Conditions

The MDR-KP strains were collected from Hangzhou First People’s Hospital, China. After the identification of genome sequencing (Personal Biotechnology Co., Ltd., China), MDR-KP was cultured in LB at 200 rpm and 37°C, and then measured at OD_600_ in a microplate reader (Tecan, Switzerland).

### MIC Determination

The MIC of antimicrobial drugs, such as cefotaxime sodium (CTX), MER, and TIG was measured by methods developed by the Clinical Laboratory Standardization Institute (CLSI). MDR-KP was incubated overnight in LB at 200 rpm. The diluted bacterial suspension and drug were added to a 96-well plate, incubated at 37°C for 18 h, and then measured at OD_600_ in a microplate reader.

### Quadrature Experiment

The quadrature experiment was designed based on the MIC of the four YHPG active ingredients (CA, Pue, Amy, and PD) set at high, medium, and low concentrations. Where the concentration of CA was 4, 8, and 16 μg/ml, the concentration of Pue was 32, 64, and 128 μg/ml; the concentration of Amy was 59.25, 118.5, and 237 μg/ml, and the concentration of PD was 15.5, 31, and 62 μg/ml. Orthogonal combinations of YHPG active ingredients are listed in Table 1.

### Killing Studies

Generally, MDR-KP was incubated overnight in LB at 200 rpm. Bacteria were then treated with different antimicrobial drug combinations or MER or their combinations and were cultured at 37°C and 200 rpm. The bacterial OD_600_ measurements were done using a microplate reader.

### Detection of Live and Dead Bacteria by Flow Cytometry

In brief, MDR-KP was diluted with PBS into LB broth containing different antimicrobial drugs or MER or their combinations at 37°C and 200 rpm. After treatment, bacteria were collected, washed, and dissolved in 1× binding buffer. Bacteria were harvested and subjected to staining with an Annexin-V/FITC and PI staining kit for 15 min in a dark environment, followed by analysis via flow cytometry. Analysis of the result was carried out using FlowJo 10.

### Antibiofilm Activity Assay

MDR-KP was diluted with PBS into LB broth containing different antimicrobial drugs or MER or their combinations at 37°C and 200 rpm overnight. After treatment, to quantify biofilm density, the biofilms were washed to remove free-floating planktonic bacteria, using 10% paraformaldehyde. Biofilms developed by adherent microorganisms on the plate were subjected to staining with crystal violet, and their absorbance was measured at 595 nm.

### Determination of AKP Activity

MDR-KP was diluted with PBS into LB broth containing different antimicrobial drugs or MER or their combinations at 37°C and 200 rpm overnight. After treatment, using the AKP kit, extracellular AKP activity at 400 nm-415 nm was detected via the microplate reader.

### qRT-PCR

MDR-KP was diluted into LB broth containing different antimicrobial drugs or MER or their combinations at 37°C and 200 rpm. After 18 h, MDR-KP was collected, the total RNA was extracted using a kit, and the total RNA purity was also measured. Quantification of mRNA was conducted by using 2X Universal SYBR Green Fast qPCR Mix. Primer sequences are listed in Table 2.

### ROS Measurement

The ROS activity of MDR-KP was assessed via quantifying the fluorescence intensity of the fluorescent probe DCFH-DA via flow cytometry. Briefly, MDR-KP was collected and the samples were incubated with a DCFH-DA probe at a dilution of 1:1000 in PBS for 30 min. Following incubation, samples were washed with PBS to remove any unbound probes. Ultimately, flow cytometry was used to indicate the levels of intracellular ROS.

### Carba NP Detection Test

Following preparation, 150 μl of NP solution (pH 7.8 ± 0.2) was added to four groups of 1.5 ml sterile centrifuge tubes (Liquid A, Liquid A with drug, Liquid B, and Liquid B with drug) into 96-well plates to treat different groups. Then, 50 μl of 1×10^8^ CFUs/ml of bacterial concentration was added, mixed, and incubated for 5-6 h. The results were recorded by observing the color change with the naked eye and by measuring the absorbance value of the mixtures at OD_492_.

### Animal Experiments

Animals and group-housed, clean-grade BALB mice (male, 20 ± 2 g) were approved by the Experimental Animal Center of Zhejiang University of Chinese Medicine and experiments were conducted in the Second-Level Biosafety Laboratory (P2) of Hangzhou First People’s Hospital affiliated with Zhejiang University. Mice were randomized into a control group, a model group, an OAY (CA, Pue, Amy, PD) group, and a YHPG 18 g/kg group. After intraperitoneal injection of 4% chloral hydrate anesthesia, the model group and drug treatment group were established via intranasal instillation of 50 μl MDR-KP (concentration of 10^9^ CFU/ml), with PBS as a control group. Mice in the OAY group were injected subcutaneously with the drug 2 h after infection (the YHPG group was administered by gavage), once, and again 12 h later, and then euthanized 24 h later. During this period, the mice were fed normally. In addition, the animal experiments were protected in accordance with the relevant regulations of the Animal Medical Center of Zhejiang University and approved by the Ethics Committee (No. 29391).

### Specimen Collection

Blood collection: mice were anesthetized with 4% chloral hydrate, the area around the eyeballs of live mice was disinfected, and the blood was collected. Lung sampling: at 12 h after the last administration, the neck was removed and lung tissue was collected for testing pathological, biological, and other indicators. Subsequently, the lung tissues were homogenized and dissolved in an extraction buffer to facilitate the analysis of malondialdehyde (MDA) and superoxide dismutase (SOD) levels utilizing designated assay kits. The concentrations of the inflammatory cytokine interleukin-1 beta (IL-1β) were then quantified by ELISA kits. The homogenate underwent centrifugation at 2,500 ×*g* for 10 min, after which the supernatant was collected. Counts of MDR-KP organisms were determined by plating serial dilutions of the lung homogenates on LB agar overnight.

### Wet-to-Dry Lung Weight Ratio

Fluid content in the lungs was measured by carefully cutting the right lung, separating the upper right lung, and then measuring its wet weight. Then, the right upper lung tissue was dried at 60°C for 24 h to measure its dry weight.

### Hematoxylin & Eosin (HE) Staining

The right middle lung tissues from different groups of mice were collected, rinsed with physiological saline, and fixed in 4% paraformaldehyde solution for 48 h. Then, the tissues were dehydrated with ethanol, embedded in paraffin, sectioned, stained with HE, and finally observed by CaseViewer software.

### Immunohistochemistry Staining

In summary, following the processes of deparaffinization and rehydration, lung tissue sections were subjected to immersion in a 1× sodium citrate buffer at 98°C for 15 min to facilitate antigen retrieval, and then treated with 3% hydrogen peroxide. Subsequently, the sections were treated with goat serum and then incubated overnight at 4°C with primary antibody against cleaved caspase-1. The sections were then incubated with secondary antibodies for 30 min. Ultimately, all sections were stained with DAB reagent and counterstained with hematoxylin. Observations of the sections were conducted utilizing an optical microscope.

### Western Blot

Lung tissues from the left side of each group of mice were collected and homogenized. Following extraction of total protein from lung tissue samples, the total protein samples were denatured and separated by SDS-PAGE, and then transferred to a PVDF membrane. The membrane was blocked with 5% BSA for 60 min. Subsequent to the washing procedure, the membrane was incubated with appropriate primary antibodies: C-GSDMD (1:1000), NLRP3 (1:1000), and NF-κB P-p65 (1:1000). After being left overnight, the secondary antibody was diluted and incubated at room temperature. Bands were exposed and visualized using enhanced chemiluminescence (ECL), and band density was quantified using Image J software.

### Statistical Analysis

All experimental results were analyzed via GraphPad. One-way analysis of variance (ANOVA) was utilized to determine any significant differences. *p* < 0.05 and *p* < 0.01 were considered statistically significant.

## Results

### Microbiological Characteristics of MDR-KP Clinical Isolates

**Fig. 1:**
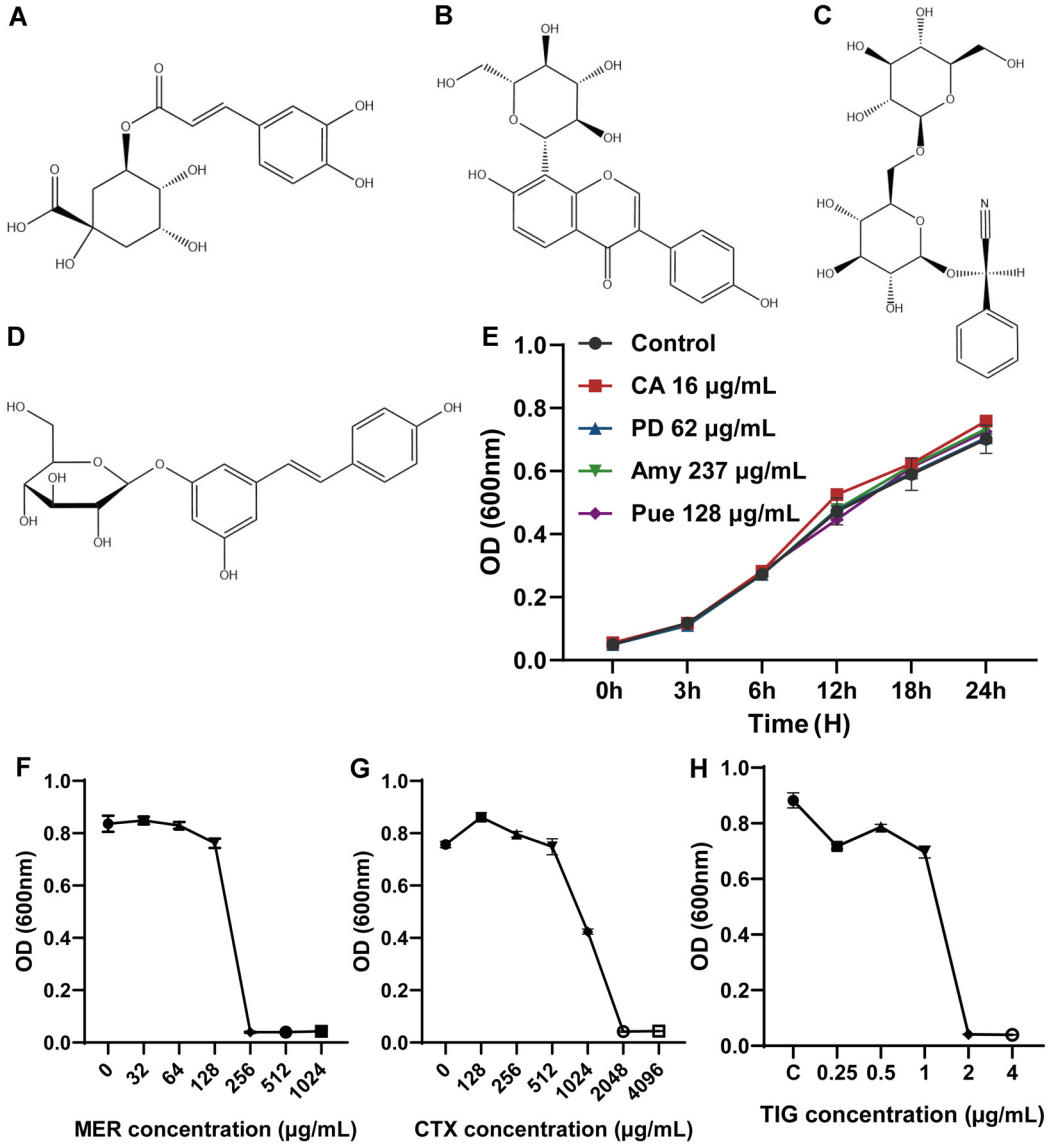
Biological characterization of bacteria. (**A**) Chemical structure of CA. (**B**) Chemical structure of Pue. (**C**) Chemical structure of Amy. (**D**) Chemical structure of PD. (**E**) Time growth curves of CA, Pue, Amy, and PD. (**F**) MIC assay of MER. (G) MIC assay of CTX. (H) MIC determination of TIG.

MDR-KP strains were resistant to MER (256 μg/ml) and CTX (2,048 μg/ml) but were sensitive to TIG (2 μg/ml)(Fig. 1F-1H). We next used the lowest harmless concentration of the CA (Fig. 1A), Pue (Fig. 1B), Amy (Fig. 1C), and PD (Fig. 1D) in our experiments. The growth rate of MDR-KP was not significantly decreased with CA, Pue, Amy, and PD (Fig. 1E).

### Antimicrobial Effects of OAY in Combination with MER on MDR-KP

**Fig. 2:**
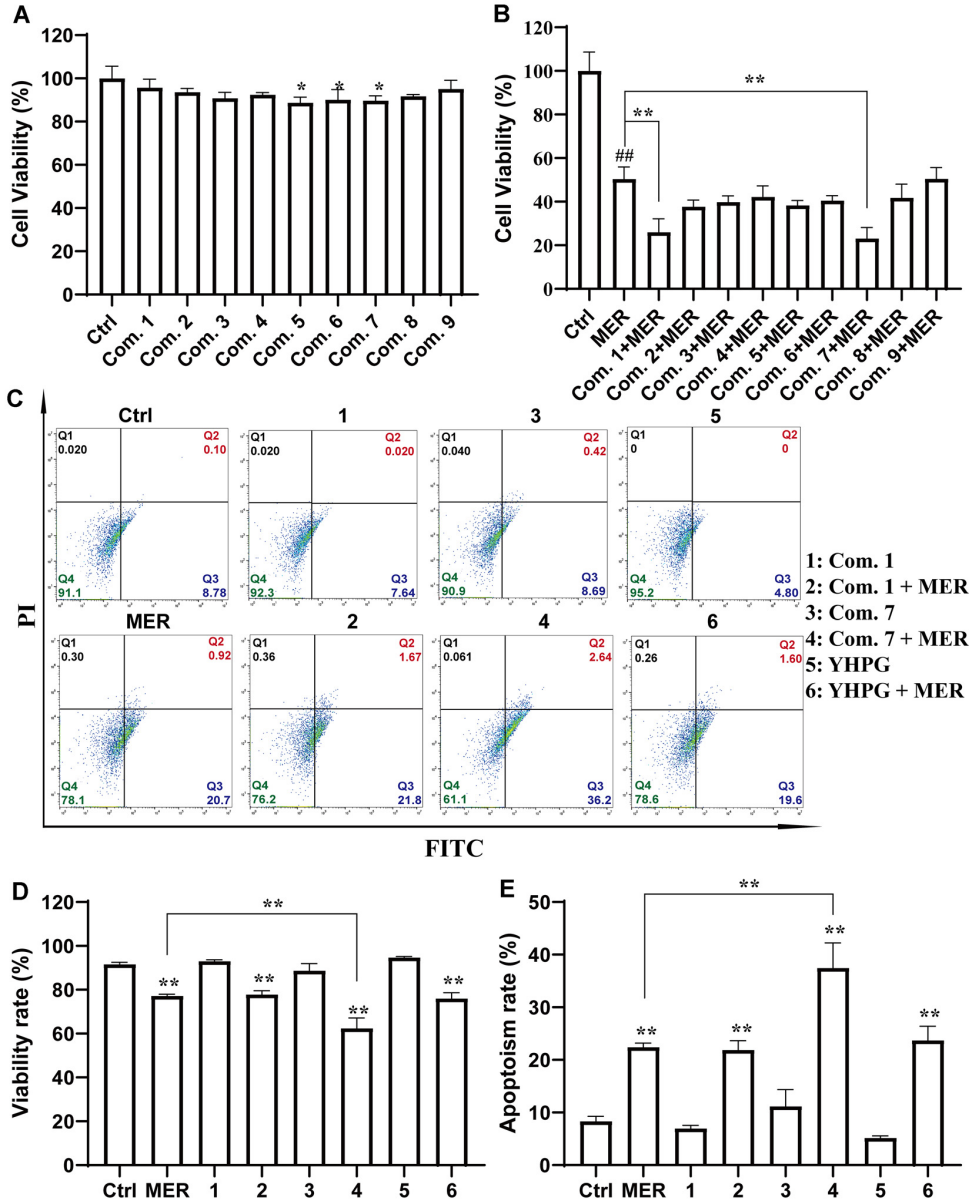
Synergistic activity of combination of OAY-MER against MDR-KP. (**A**) Activity assay of OAY dosing (**B**) Activity assay of OAY dosing in combination with MER. (**C-E**) Apoptosis-like results. 1: Com. 1; 2: Com. 1 + MER; 3: Com. 7; 4: Com. 7 + MER; 5: YHPG; 6: YHPG + MER. *p < 0.05 vs. Ctrl group, **p < 0.01 vs. Ctrl group.

There was a significant difference when OAY was combined with MER compared to the control (Fig. 2A and 2B). By matching Annexin-V to PI, it was possible to distinguish between live and apoptotic-like, dead bacteria. As shown in Fig. 2D and 2E, apoptosis-like condition increased and survival decreased with MER in combination with OAY groups. Taken together, OAY could compromise bacterial structure and lead to an apoptosis-like appearance of MDR-KP.

### The OAY/YHPG-MER Combination Showed Synergistic Activity against tmexCD1-*toprJ1*-Carrying Bacteria and Promoted ROS

**Fig. 3:**
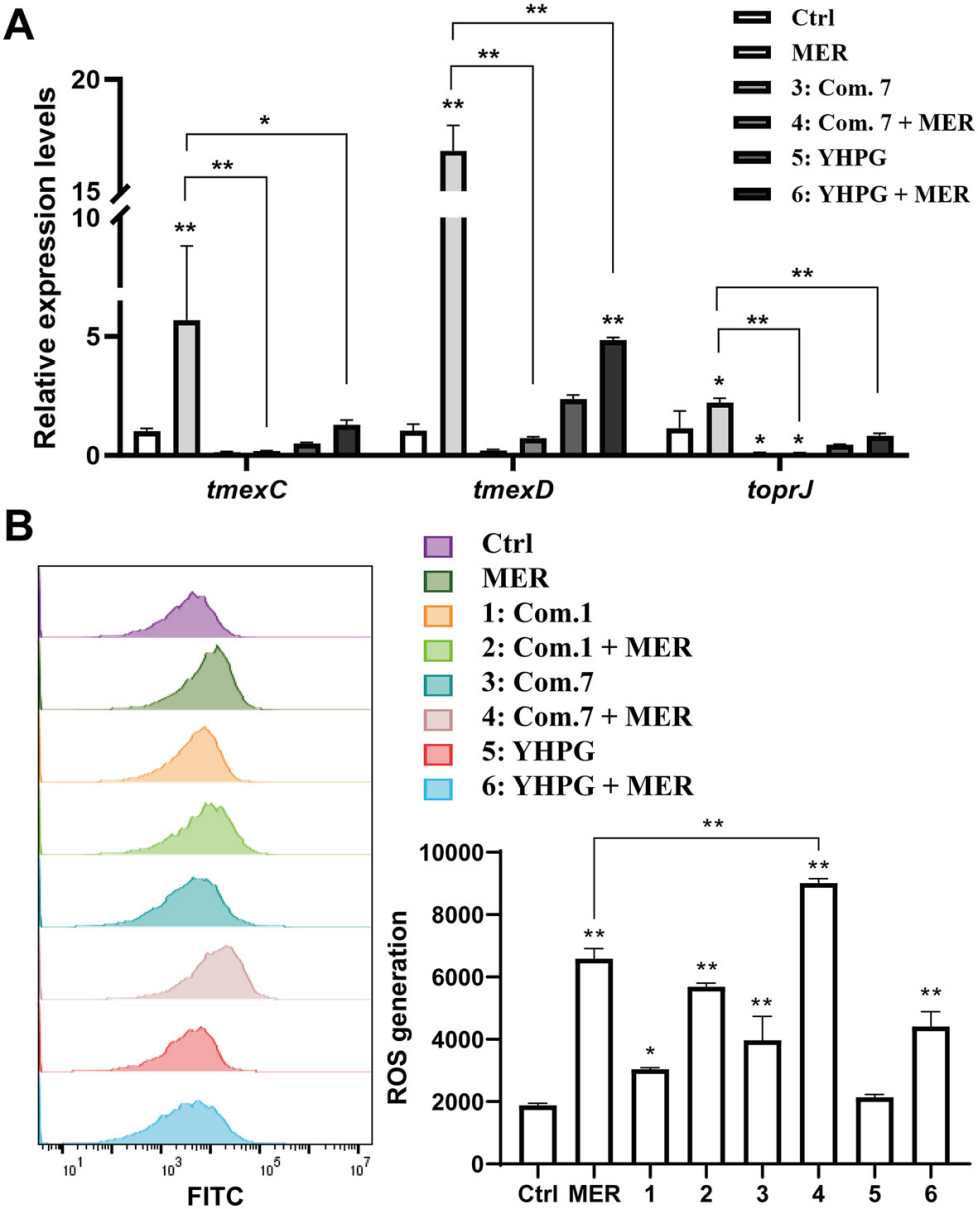
OAY/YHPG-MER combination exerted synergy inhibiting the expression of tmexCD1-*toprJ1* and increasing ROS, which damaged the bacterial wall, leading to apoptosis-like. (**A**) Relative expression of *tmexC1*, *tmexD1*, and *toprJ1* in the presence of MER (64 μg/ml), OAY, and their combinations. (**B**) ROS. 1: Com. 1; 2: Com. 1 + MER; 3: Com. 7; 4: Com. 7 + MER; 5: YHPG; 6: YHPG + MER. *p < 0.05 vs. Ctrl group, **p < 0.01 vs. Ctrl group.

Having demonstrated that OAY enhanced the bactericidal effect of MER, we sought to understand the underlying mechanisms. First, we determined the gene expression in efflux pump tmexCD1-*toprJ1* by qPCR. As shown in Fig. 3A, compared with MER group, the expression of *tmexC1*, *tmexD1*, and *toprJ1* was significantly inhibited by MER combined with OAY/YHPG. As shown in Fig. 3B, the intensity of OAY/YHPG-MER combinations-induced intracellular fluorescence of the bacteria was enhanced. These results suggested that OAY/YHPG-MER combinations could block the RND efflux pump and promote oxidative stress, resulting in the intracellular bacteria accumulation of MER.

### OAY/YHPG-MER Damaged the Bacterial Membrane of MDR-KP

**Fig. 4:**
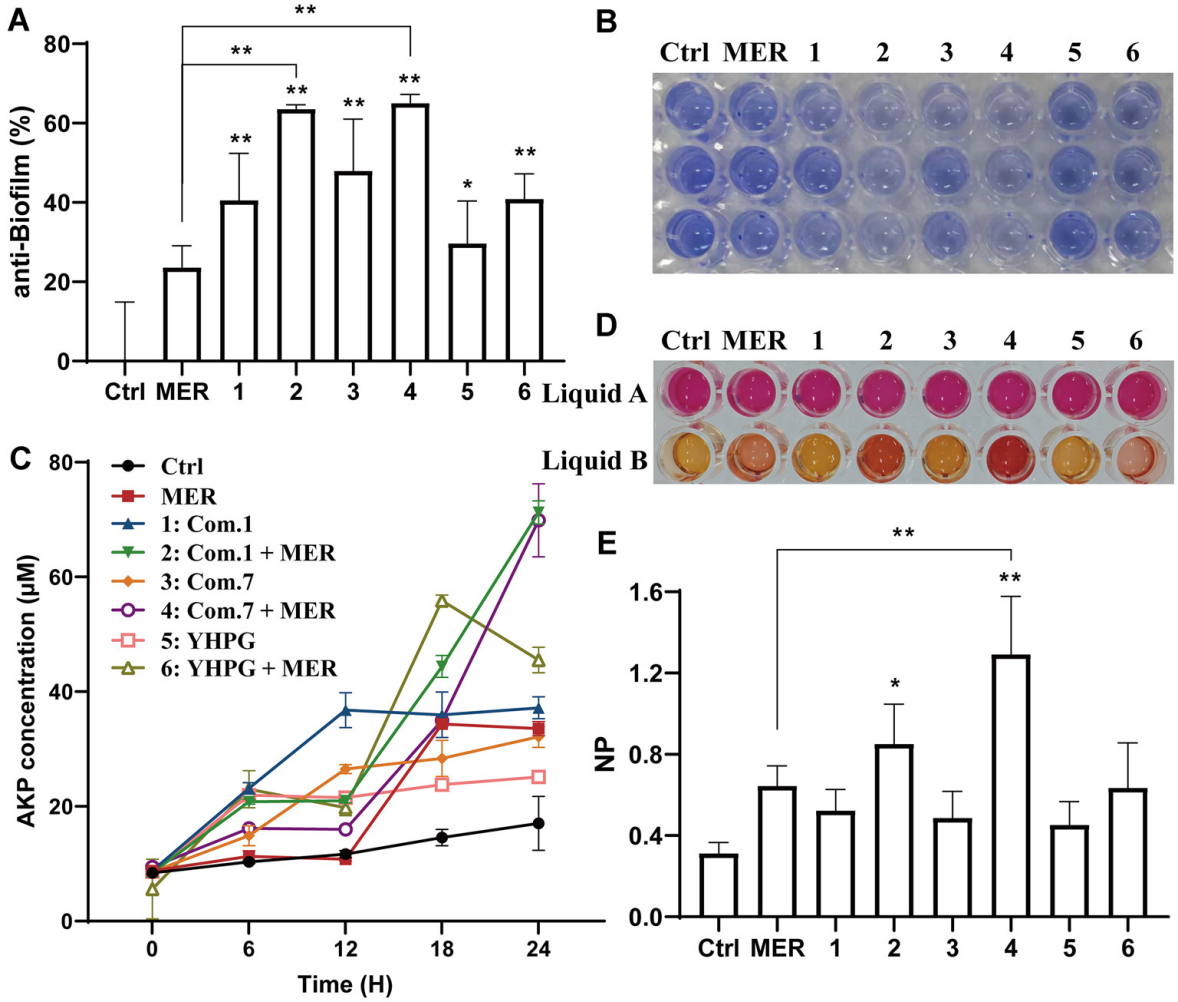
Effects of OAY/YHPG on MDR-KP biofilm formation. Crystal violet staining of MDR-KP biofilms treated with OAY/YHPG -MER combination (**A-B**). (**C**) AKP activity of MDR-KP. (**D, E**) NP activity of MDR-KP. 1: Com. 1; 2: Com. 1 + MER; 3: Com. 7; 4: Com. 7 + MER; 5: YHPG; 6: YHPG + MER. *p < 0.05 vs. Ctrl group, **p < 0.01 vs. Ctrl group.

The elevated expression of ROS resulting from the OAY/YHPG-MER combination subsequently enhanced the permeability of the bacterial membrane, ultimately causing bacterial death. To confirm this, we analyzed the OAY/YHPG-MER combination influence in MDR-KP biofilm formation capacity by crystal violet staining assay. As shown in Fig. 4B, following staining with crystalline violet, a clear color gradient was observed. The AKP activity in the untreated control group showed no significant change within each time point. Meanwhile, the AKP activity in the treated group showed a time-dependent increase, and particularly, the highest activity was observed in the OAY/YHPG group in combination with MER. As shown in Fig. 4D and 4E, by analyzing the color change (Fig. 4D) and the absorbance value of the samples out of 492 nm (Fig. 4E), we found that the color turned to red in the wells of the OAY-MER combination co-culture, and the OD_492_ nm value of the combined group changed significantly, which suggested that OAY in combination with MER inhibited carbapenemase production by MDR-KP. This result showed that the OAY/YHPG -MER combination via inducing ROS production caused destruction of the permeability of bacterial wall, ultimately contributing to bacterial death.

### OAY (Com.7) and YHPG Ameliorated MDR-KP-Induced Lung Injury in Mice

**Fig. 5:**
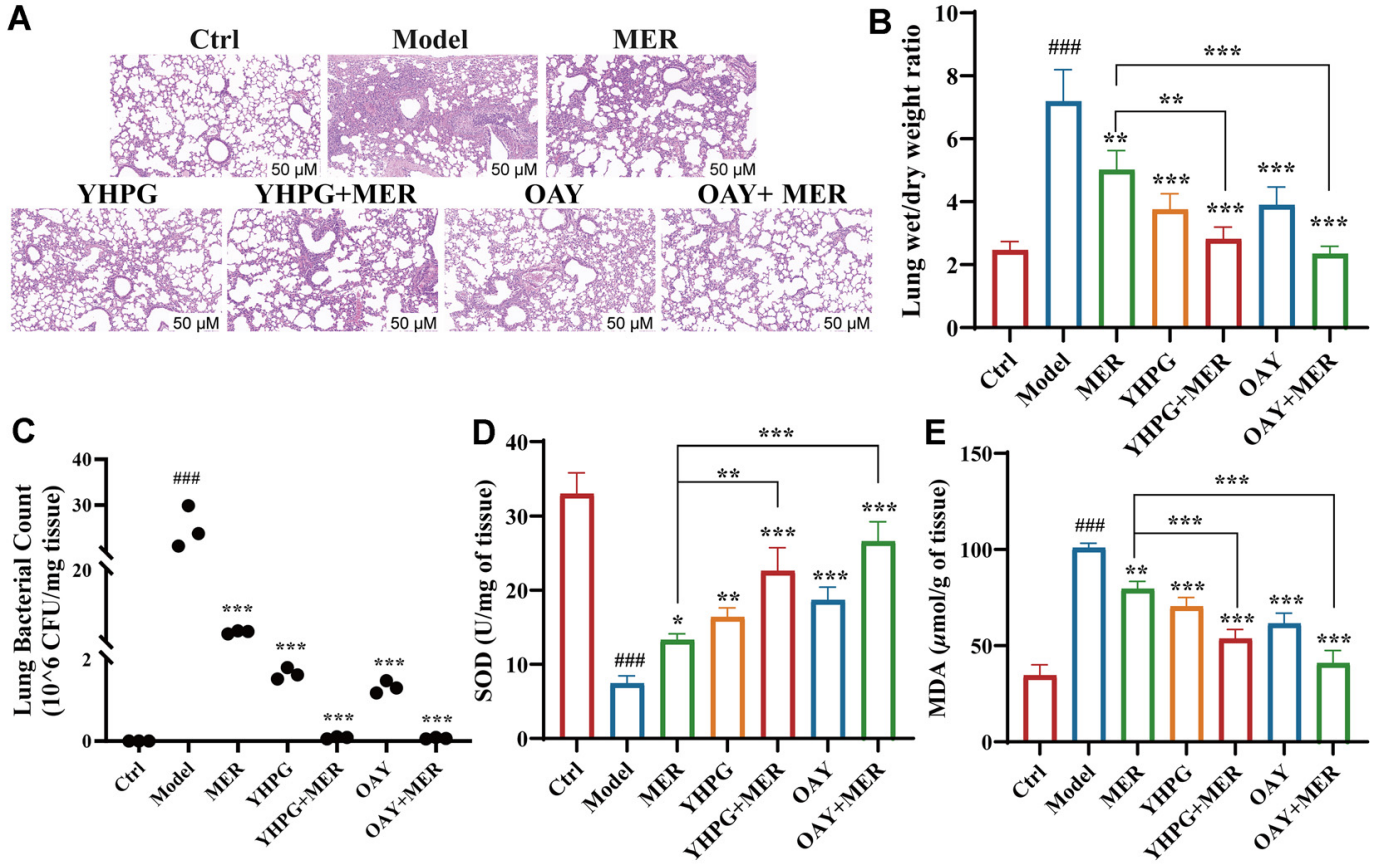
OAY (Com.7)/YHPG alleviated the pathological characteristics of lung induced in mice by MDR-KP. (**A**) The images of lungs in different groups, with HE staining. (**B**) Statistical results of W/D in different groups. (**C**) Lung bacterial content changes after MDR-KP infection. Effects of YHPG and OAY on SOD (**D**), and MDA (**E**) levels in lung tissues after MDR-KP infection. OAY: Com. 7; #*p* < 0.05 vs. Ctrl group, **p < 0.01 vs. Model group.

Briefly, we found the bacterial inhibition effect of Com.7 was superior to that of Com.1, and therefore we used Com.7 for subsequent in vivo animal experiments. To investigate the impact of OAY (Com.7) on lung injury induced by MDR-KP, we performed HE analysis and evaluated lung W/D in mice lung tissues. In comparison to the control group, the MDR-KP infection led to significant lung injury. Conversely, treatment with OAY and YHPG markedly ameliorated the lung injury induced by MDR-KP, as evidenced by a reduction in both intra-alveolar and interstitial edema (Fig. 5A). The W/D mass ratio was significantly higher in the model group, suggesting that MDR-KP infection induced pulmonary edema in mice. In contrast, drug treatment improved MDR-KP-induced pulmonary edema. We therefore concluded that OAY and YHPG could therapeutically attenuate pulmonary edema induced by MDR-KP in mice (Fig. 5B). We also assessed lung infections in mice by examining lung tissue for levels of bacteria. As illustrated in Fig. 5C, the concentration of MDR-KP was significantly elevated in the model group, and by contrast, the groups that received pretreatment with YHPG and OAY exhibited a diminished MDR-KP content in the lung tissue. The bacterial load was greatly declined in the YHPG+MER and OAY+MER treatment groups compared to the MER group. These findings indicated that YHPG and OAY exerted considerable effects in reducing the levels of MDR-KP in the lung. As shown in Fig. 5D and 5E, MDR-KP significantly elevated MDA levels and reduced SOD levels compared to the control group, while YHPG and OAY treatment reversed this effect.

### OAY (Com.7) and YHPG Inhibited the Activation of Pyroptosis via the NF‐κB/NLRP3 Pathway in MDR-KP-Infected Mice

**Fig. 6:**
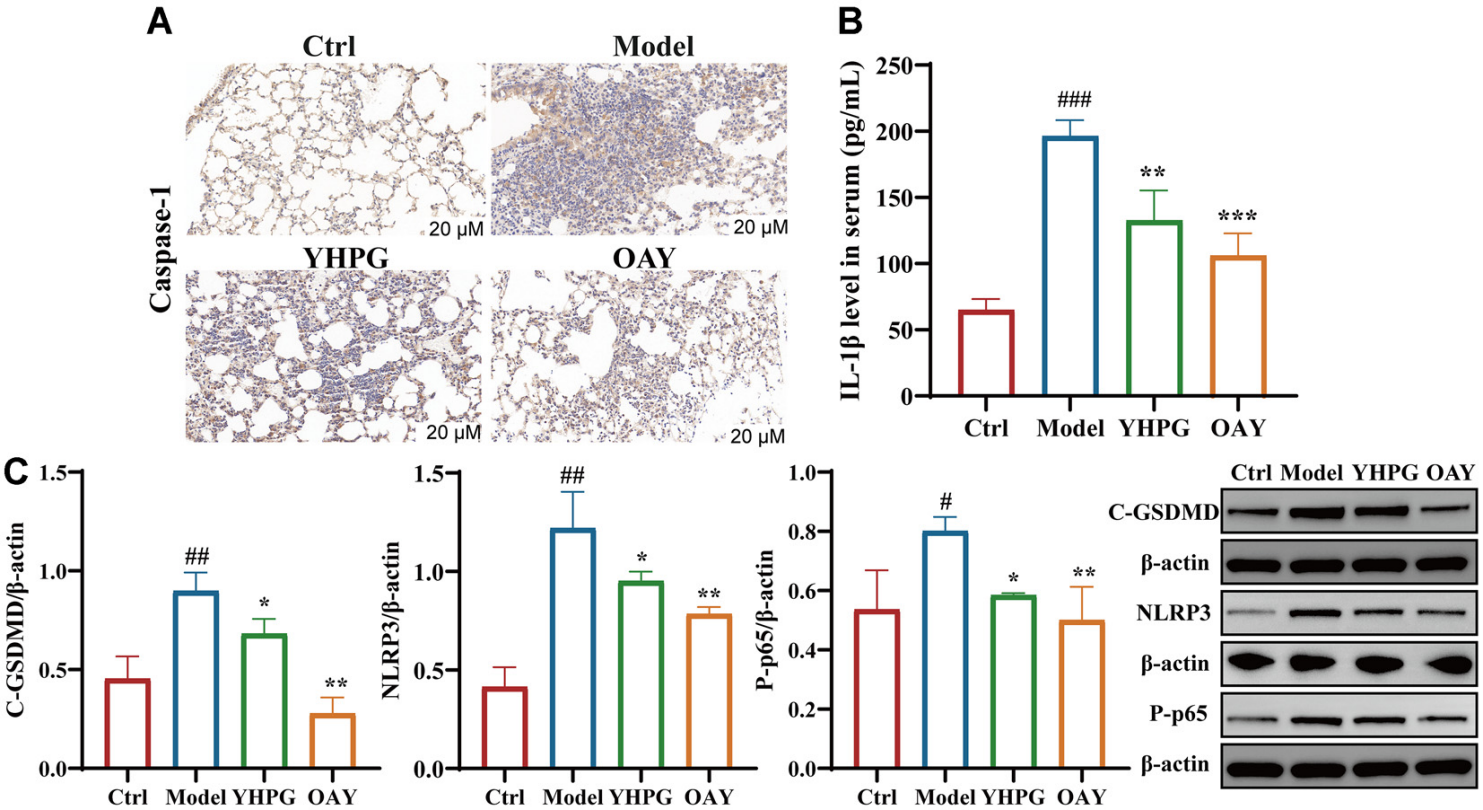
OAY (Com.7) and YHPG reduced lung injury in MDR-KP-infected mice by attenuating GSDMD/ NLRP3/NF-κB expression. (**A**) Immunohistochemical staining was used to detect the protein expressions of caspase-1. (**B**) Effects of YHPG and OAY on IL-1β production and secretion in mice from MDR-KP-induced pneumonia mice. (**C**) The protein levels of NLRP3, NF-κB-P-p65, and C-GSDMD were detected by western blotting, and the grayscale values of the bands were analyzed using ImageJ software. ^#^*p* < 0.05 vs. Ctrl group, ^##^*p* < 0.01 vs. Ctrl group, *p < 0.05 vs. Model group, **p < 0.01 vs. Model group.

MDR-KP mice exhibited a much higher protein level of caspase-1, compared with control mice, while YHPG and OAY treatment reversed this effect (Fig. 6A). The levels of IL-1β were markedly elevated following MDR-KP infection in comparison to the control group, while YHPG and OAY treatment reversed this effect (Fig. 6B). Then, we assessed the expression level of pivotal proteins of the inflammatory pathway (NF‐κB/NLRP3, GSDMD). Results demonstrated that MDR-KP infection significantly elevated the expression of NF‐κB, NLRP3, and GSDMD. OAY and YHPG groups both successfully inhibited the activation of NLRP3 and P-p65 induced by MDR-KP infection. The above data suggested that OAY and YHPG mainly attenuate MDR-KP-induced lung injury via suppression of the NF‐κB/NLRP3 pathway.

## Discussion

Antimicrobial resistance represents a significant challenge to global health and seriously jeopardizes patient lives [36]. The integration of antibiotics with non-antibiotic agents that may either impede bacterial resistance mechanisms or augment the efficacy of antibiotics presents a viable and sustainable approach to addressing the challenge posed by multidrug-resistant bacteria [36]. We observed that the combination of OAY/YHPG-MER effectively counteracted the MER resistance mediated by tmexCD-toprJ, thereby underscoring the efficacy of this drug combination with our research. The efflux pump tmexCD-toprJ gene cluster underwent horizontal gene transfer between bacteria via plasmids, a mechanism that enabled the rapid spread of drug-resistant genes in bacterial populations, allowing bacteria to exhibit multi-drug resistance to quinolones, fluoroquinolones, aminoglycosides, and tetracycline antibiotics [17]. Previous studies have shown that BEN, a non-steroidal anti-inflammatory drug, reversed tmexCD-toprJ-mediated TIG resistance and significantly enhanced TIG antimicrobial activity against drug-resistant KP and *P. mirabilis* [37]. In this study, the expression of *tmexC1*, *tmexD1*, and *toprJ1* in MDR-KP was significantly inhibited by MER combined with OAY/YHPG. Consequently, the combination of antibiotics with non-antibiotic agents presents a novel strategy to deal with the bacterial resistance situation.

Oxidative stress induced by ROS, a novel antibacterial strategy, has the potential to inflict damage on nucleic acids, proteins, and lipids, disrupt the bacterial oxidative milieu, and ultimately lead to bacterial death [18, 38-40]. ROS production contributed to the bacteriostatic effects of antibiotics [18]. In our research, the findings indicated that OAY also promoted the accumulation of ROS. In addition, the OAY/YHPG-MER combination inhibited biofilm formation, which subsequently promoted an increased uptake of MER, thereby augmenting the killing of MDR-KP bacteria. AKP was often used to reflect the integrity of the bacterial outer membrane and cell wall [41-43]. It had been reported that the outer membrane of *E. coli*, which dealt with oregano essential oil, was disrupted and extracellular AKP activity increased [43]. This result was similar to our findings; the higher AKP activity of bacteria was increased with the combination of OAY/YHPG-MER, which indicated that the MDR-KP outer membrane had been damaged. Overall, the combination of OAY/YHPG-MER induced oxidative stress in bacteria, disrupting the integrity of the outer membrane and cell wall. Meanwhile, the increased permeability of the outer membrane led to MER accumulation, and ultimately to bacterial death. In conclusion, combination therapy using OAY/YHPG-MER has enormous potential to restore antibiotic efficacy in the clinical setting.

Numerous signaling pathways contribute to the inflammatory response, and NF-κB is recognized as a critical component [44]. Activated NF‐κB plays a significant role in modulating cell proliferation and apoptosis, as well as in the synthesis of inflammatory cytokines [7, 8, 44]. LPS was known to activate the TLR4/NF-κB signaling pathway and initiate transcription of its downstream inflammatory cytokines IL-6, TNFa, and chemokines [45, 46]. In this research, our results indicated that MDR-KP markedly increased the expression levels of NF-κB, while OAY and YHPG decreased the expression of NF-κB in MDR-KP-infected mice. NLRP3 inflammasome, one of the most essential steps in host defense, is activated by NF-κB, thereby upregulating downstream target proteins caspase-1, pro-IL-18, as well as pro-IL-18 expression [46, 47]. In this research, lung tissues from mice subjected to exposure to MDR-KP exhibited elevated NLRP3 and caspase-1 as well as cleaved GSDMD expression, while YHPG and OAY treatment reversed this effect. In conclusion, the therapeutic potential of OAY in addressing pyroptosis in lung injury may be related to the activation of the NLRP3 inflammasome.

**Fig. 7:**
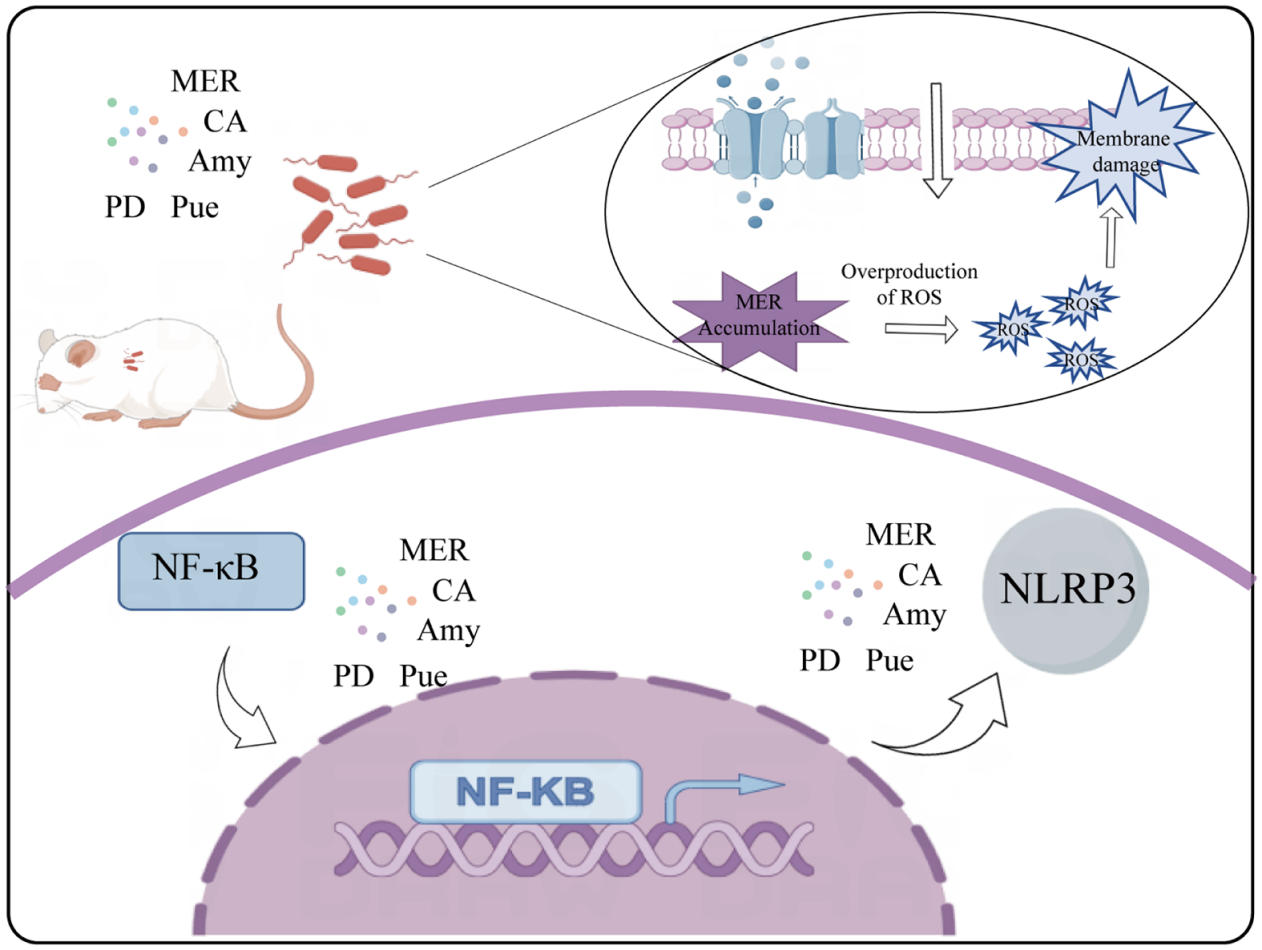
A schematic diagram showed that the OAY when combined with MER reversed tmexCD1-topJ1-mediated MER resistance in MDR-KP and inhibited NF-κB pathway and NLRP3 inflammasome activation.

Our study showed the combination of OAY/YHPG-MER as a promising antimicrobial extract that potentially increases the susceptibility of resistant strains to antibiotics. As shown in Fig. 7, we also propose that the OAY/YHPG-MER combination disrupts the overall integrity of the bacterial membrane through tmexCD-toprJ and oxidative stress, leading to enhanced cell wall permeability and thereby contributing to the accumulation of antimicrobial drugs in bacteria, ultimately leading to bacterial death. Furthermore, OAY could ameliorate the pathological process of lung injury due to MDR-KP infection, which might be by inhibiting the NF-κB pathway and activating NLRP3 inflammasome. In conclusion, a combination of OAY/YHPG-MER demonstrated significant potential for enhancing the effectiveness of antibiotics within clinical applications and our study provides a platform for future research.

## Conclusion

In conclusion, these data suggested that a combination of OAY/YHPG-MER was a promising treatment method for addressing the resistance conferred by novel RND efflux pumps to MER. However, there were still several limitations in this study. First, no tmexCD-toprJ pump inhibition experiments were done, and the effect of the drug on the upstream genes of tmexCD-toprJ was not clarified. Second, specific targets regarding NF-κB/NLRP3 signaling pathways were not studied in depth. Finally, the specific mechanism of the drug has not been elucidated, and additional preclinical studies are necessary to thoroughly investigate the therapeutic potential of this drug combination.

## Figures and Tables

**Table 1:** Orthogonal combinations of YHPG active ingredients table.

Number	CA (μg/ml)	Pue (μg/ml)	Amy (μg/ml)	PD (μg/ml)
Com.1	16	128	237	62
Com.2	16	64	59.25	31
Com.3	16	32	118.5	15.5
Com.4	8	32	59.25	62
Com.5	8	128	118.5	31
Com.6	8	64	237	15.5
Com.7	4	64	118.5	62
Com.8	4	32	237	31
Com.9	4	128	59.25	15.5

**Table 2:** The primer sequences for qRT-PCR.

Gene	Forward (5'-3')	Reverse (5'-3')
*tmexC1*	GTGCGAGCGACCAGC	TTGAGCCCCTGCGGC
*tmexD1*	CGTCAACAACGCCAT	CGCGAGCCGTCCTG
*toprJ1*	CCGACTACGAAGGCA	CGCAGGTAGTCGTCC
16S rRNA	TTGACGTTACCCGCAGAAGAA	GCTTGCACCCTCCGTATTACC

## References

[1] Martin RM, Bachman MA (2018). Colonization, infection, and the accessory genome of *Klebsiella pneumoniae*. Front. Cell. Infect. Microbiol..

[2] Lan P, Jiang Y, Zhou J, Yu Y (2021). A global perspective on the convergence of hypervirulence and carbapenem resistance in *Klebsiella pneumoniae*. J. Glob. Antimicrob. Resist..

[3] Aksomaitiene J, Novoslavskij A, Kudirkiene E, Gabinaitiene A, Malakauskas M (2020). Whole genome sequence-based prediction of resistance determinants in high-level multidrug-resistant *Campylobacter jejuni* isolates in Lithuania. Microorganisms.

[4] Raza A, Ngieng SC, Sime FB, Cabot PJ, Roberts JA, Popat A (2021). Oral meropenem for superbugs: challenges and opportunities. Drug Discov. Today.

[5] Slama TG (2008). Clinical review: balancing the therapeutic, safety, and economic issues underlying effective antipseudomonal carbapenem use. Crit. Care.

[6] Liu Q, Ma H, Sun X, Liu B, Xiao Y, Pan S (2019). The regulatory ZFAS1/miR-150/ST6GAL1 crosstalk modulates sialylation of EGFR via PI3K/Akt pathway in T-cell acute lymphoblastic leukemia. J. Exper. Clin. Cancer Res.

[7] Ruland J (2011). Return to homeostasis: downregulation of NF-κB responses. Nat. Immunol..

[8] Lai JL, Liu YH, Liu C, Qi MP, Liu RN, Zhu XF (2017). Indirubin inhibits LPS-induced inflammation *via* TLR4 abrogation mediated by the NF-kB and MAPK signaling pathways. Inflammation.

[9] Wang X, Liu M, Geng N, Du Y, Li Z, Gao X (2022). *Staphylococcus aureus* mediates pyroptosis in bovine mammary epithelial cell via activation of NLRP3 inflammasome. Vet. Res..

[10] Shi J, Zhao Y, Wang K, Shi X, Wang Y, Huang H (2015). Cleavage of GSDMD by inflammatory caspases determines pyroptotic cell death. Nature.

[11] Kesavardhana S, Malireddi RKS, Kanneganti TD (2020). Caspases in cell death, inflammation, and pyroptosis. Ann. Rev. Immunol..

[12] Shan M, Wan H, Ran L, Ye J, Xie W, Lu J (2024). Dynasore alleviates LPS-induced acute lung injury by inhibiting NLRP3 inflammasome-mediated pyroptosis. Drug Des. Devel. Ther..

[13] Jorgensen SCJ, Rybak MJ (2018). Meropenem and Vaborbactam: stepping up the battle against Carbapenem-resistant Enterobacteriaceae. Pharmacotherapy.

[14] Martínez-Martínez L, Pascual A, Hernández-Allés S, Alvarez-Díaz D, Suárez AI, Tran J (1999). Roles of beta-lactamases and porins in activities of carbapenems and cephalosporins against *Klebsiella pneumoniae*. Antimicrob. Agents Chemother..

[15] Lv L, Wan M, Wang C, Gao X, Yang Q, Partridge SR (2020). Emergence of a plasmid-encoded resistance-nodulation-division efflux pump conferring resistance to multiple drugs, including tigecycline, in *Klebsiella pneumoniae*. mBio.

[16] Xiao X, Huan Q, Huang Y, Liu Y, Li R, Xu X (2022). Metformin reverses tmexCD1-toprJ1- and tet(A)-mediated high-level tigecycline resistance in *K. pneumoniae*. Antibiotics (Basel, Switzerland).

[17] Wu Y, Zhuang Y, Wu C, Jia H, He F, Ruan Z (2024). Global emergence of Gram-negative bacteria carrying the mobilised RND-type efflux pump gene cluster tmexCD-toprJ variants. Lancet Microbe.

[18] Lam PL, Wong RS, Lam KH, Hung LK, Wong MM, Yung LH (2020). The role of reactive oxygen species in the biological activity of antimicrobial agents: an updated mini review. Chem. Biol. Interact..

[19] Ezraty B, Gennaris A, Barras F, Collet JF (2017). Oxidative stress, protein damage and repair in bacteria. Nat. Rev. Microbiol..

[20] Qi X, Zhang Y, Guo H, Hai Y, Luo Y, Yue T (2020). Mechanism and intervention measures of iron side effects on the intestine. Crit. Rev. Food Sci. Nutr..

[21] Vuotto C, Longo F, Pascolini C, Donelli G, Balice MP, Libori MF (2017). Biofilm formation and antibiotic resistance in *Klebsiella pneumoniae* urinary strains. J. Appl. Microbiol..

[22] Rahdar HA, Malekabad ES, Dadashi AR, Takei E, Keikha M, Kazemian H (2019). Correlation between biofilm formation and carbapenem resistance among clinical isolates of *Klebsiella pneumoniae*. Ethiop. J. Health Sci..

[23] Zhang W, He M, Kong N, Niu Y, Li A, Yan Y (2024). Study on the inhibition activity and mechanism of Tanreqing against *Klebsiella pneumoniae* biofilm formation in vitro and in vivo. Front. Cell. Infect. Microbiol..

[24] Vestby LK, Grønseth T, Simm R, Nesse LL (2020). Bacterial biofilm and its role in the pathogenesis of disease. Antibiotics (Basel, Switzerland).

[25] Li L, Gao X, Li M, Liu Y, Ma J, Wang X (2024). Relationship between biofilm formation and antibiotic resistance of *Klebsiella pneumoniae* and updates on antibiofilm therapeutic strategies. Front. Cell. Infect. Microbiol..

[26] Singh A, Amod A, Pandey P, Bose P, Pingali MS, Shivalkar S, *et al*. 2022. Bacterial biofilm infections, their resistance to antibiotics therapy and current treatment strategies. *Biomed. Mater.* **17.** doi: 10.1088/1748-605X/ac50f6. 10.1088/1748-605X/ac50f6 35105823

[27] Ribeiro SM, Cardoso MH, Cândido Ede S, Franco OL (2016). Understanding, preventing and eradicating *Klebsiella pneumoniae* biofilms. Future Microbiol..

[28] Song M, Liu Y, Huang X, Ding S, Wang Y, Shen J (2020). A broad-spectrum antibiotic adjuvant reverses multidrug-resistant Gram-negative pathogens. Nat. Microbiol..

[29] Liu W, Chen G, Dou K, Yi B, Wang D, Zhou Q (2023). Eugenol eliminates carbapenem-resistant *Klebsiella pneumoniae* via reactive oxygen species mechanism. Front. Microbiol..

[30] Guan X, Jin L, Yu D, He Y, Bao Y, Zhou H (2023). Glycyrrhetinic acid prevents carbapenem-resistant *Klebsiella pneumoniae*induced cell injury by inhibiting mitochondrial dysfunction via Nrf-2 pathway. Microb. Pathog..

[31] Guan X, Jin L, Zhou H, Chen J, Wan H, Bao Y (2023). Polydatin prevent lung epithelial cell from *Carbapenem-resistant*
*Klebsiella pneumoniae* injury by inhibiting biofilm formation and oxidative stress. Sci. Rep..

[32] Mattick JS, Amaral PP, Carninci P, Carpenter S, Chang HY, Chen LL (2023). Long non-coding RNAs: definitions, functions, challenges and recommendations. Nat. Rev. Mol. Cell Biol..

[33] Lu C, Jin L, Zhou H, Yang J, Wan H (2024). Chlorogenic acid inhibits macrophage PANoptosis induced by cefotaxime-resistant *Escherichia coli*. Arch. Microbiol..

[34] Peng XQ, Zhou HF, Lu YY, Chen JK, Wan HT, Zhang YY (2016). Protective effects of *Yinhuapinggan granule* on mice with influenza viral pneumonia. Int. Immunopharmacol..

[35] Yan X, Jin L, Zhou H, Wan H, Wan H, Yang J (2023). Amygdalin reverses macrophage PANoptosis induced by drug-resistant *Escherichia coli*. J. Microbiol. Biotechnol..

[36] Liu Y, Tong Z, Shi J, Li R, Upton M, Wang Z (2021). Drug repurposing for next-generation combination therapies against multidrugresistant bacteria. Theranostics.

[37] Tong Z, Xu T, Deng T, Shi J, Wang Z, Liu Y (2021). Benzydamine reverses TMexCD-TOprJ-mediated high-level tigecycline resistance in gram-negative bacteria. Pharmaceuticals (Basel, Switzerland).

[38] Brynildsen MP, Winkler JA, Spina CS, MacDonald IC, Collins JJ (2013). Potentiating antibacterial activity by predictably enhancing endogenous microbial ROS production. Nat. Biotechnol..

[39] Sun L, Sun L, Li X, Hu X, Wang X, Nie T (2021). A novel tigecycline adjuvant ML-7 reverses the susceptibility of tigecyclineresistant *Klebsiella pneumoniae*. Front. Cell. Infect. Microbiol..

[40] Dharmaraja AT (2017). Role of Reactive Oxygen Species (ROS) in therapeutics and drug resistance in cancer and bacteria. J. Med. Chem..

[41] Yang SK, Yusoff K, Thomas W, Akseer R, Alhosani MS, Abushelaibi A (2020). Lavender essential oil induces oxidative stress which modifies the bacterial membrane permeability of carbapenemase producing *Klebsiella pneumoniae*. Sci. Rep..

[42] Perez AP, Perez N, Lozano CMS, Altube MJ, de Farias MA, Portugal RV (2019). The anti MRSA biofilm activity of *Thymus vulgaris* essential oil in nanovesicles. Phytomed. Int. J. Phytother. Phytopharmacol..

[43] Chen Y, Zhao J, Liu C, Wu D, Wang X (2023). In-vitro antibacterial activity and mechanism of *Monarda didyma* essential oils against *Carbapenem-resistant*
*Klebsiella pneumoniae*. BMC Microbiol..

[44] Ma M, Pei Y, Wang X, Feng J, Zhang Y, Gao MQ (2019). LncRNA XIST mediates bovine mammary epithelial cell inflammatory response via NF-κB/NLRP3 inflammasome pathway. Cell Prolif..

[45] Sun SC (2017). The non-canonical NF-κB pathway in immunity and inflammation. Nat. Rev. Immunol..

[46] Zhang C, Wang X, Wang C, He C, Ma Q, Li J (2021). Qingwenzhike prescription alleviates acute lung injury induced by LPS via inhibiting TLR4/NF-kB pathway and NLRP3 inflammasome activation. Front. Pharmacol..

[47] McVey MJ, Steinberg BE, Goldenberg NM (2021). Inflammasome activation in acute lung injury. Am. J. Physiol. Lung Cell. Mol. Physiol..

